# Gender Equality Training for Students in Higher Education: Protocol for a Scoping Review

**DOI:** 10.2196/44584

**Published:** 2023-09-20

**Authors:** Claire Condron, Mide Power, Midhun Mathew, Siobhan M Lucey

**Affiliations:** 1 RCSI SIM RCSI University of Medicine and Health Sciences Dublin Ireland; 2 Cork University Dental School & Hospital Cork Ireland

**Keywords:** gender equality, student leaders, simulation-based education, communications skills, training, higher education, sustainable goal, scoping, review method, PRISMA, Preferred Reporting Items for Systematic Reviews and Meta-Analyses, search strategy, gender, equality, equalities, inclusion, diversity, postsecondary, student, teaching, coaching, teacher, educator

## Abstract

**Background:**

The principles of gender equality are integral to the goals, targets, and indicators of all sustainable development goals. Higher education institutes can be powerful agents for promoting gender equality, diversity, and inclusion not only in the higher education context but also in society as a whole. To address and overcome gender inequality in the higher education environment, experts posit that change needs to occur from day 1 of the student’s academic experience. To this end, training is required. A preliminary review of the literature indicates that multiple gender equality–based training programs or initiatives for students have been designed and evaluated in second and third-level education settings. Examples of educational activities undertaken include delivery of didactic teaching, participation in a face-to-face collaboration project, site visits, case studies, and coaching. Yet, our initial search indicated that, to date, a comprehensive review collating the available evidence on gender equality training for third-level students has not yet been carried out.

**Objective:**

Our review seeks to identify and explore the existing literature on gender equality training interventions for third-level students, with a particular emphasis on training content, methodology, and outcome evaluation.

**Methods:**

This scoping review will be structured using the Arskey and O’Malley’s 5-stage framework and will consider empirical research and other relevant published works that address gender equality training. Systematic searches will be carried out in 6 research databases and the gray literature using key search terms. Inclusion and exclusion criteria have been defined, and a data charting tool created to methodically extract information from selected literature. The free web software Rayyan will be used for primary screening where each reference will be screened in duplicate first by title, then abstract, and finally by full text.

**Results:**

This review forms part of the LIBRA (Balance) study and has received peer-reviewed grant funding from the Irish Higher Education Authority. LIBRA aims to use simulation-based education to develop a gender equality leadership training program for student leaders in higher education. The findings will be summarized in tabular form, and a narrative synthesis produced to inform curriculum development.

**Conclusions:**

This review seeks to inform curriculum design by reporting on the gender equality–enabling skills and leadership skills necessary to foster gender equality. This paper should inform recommendations for training and catalyze future research in this rapidly evolving area.

**International Registered Report Identifier (IRRID):**

DERR1-10.2196/44584

## Introduction

### Background

Higher education institutions (HEIs) can be powerful institutions for promoting gender equality, diversity, and inclusion not only in the higher education context but also in society in general; however, universities remain both gendered and gendering organizations [1]. Recent statistical reports on gender equality data in the European Union show that female students make up the majority of the undergraduate population in HEIs, yet several published reports highlight ongoing cultural, institutional, and structural barriers inhibiting gender equality from true realization in this sector [2]. Despite the global feminization of the third-level student population, women are not progressing at the same rate as men in their academic careers. Male students remain a majority among postgraduates [2] and at leadership level, women accounted for only 24% of professorial chairs and 22% of heads of institutions in the higher education sector across the European Union in 2017 [1]. This gender imbalance is replicated in senior leadership in Irish third-level institutions [3,4].

Institutional structures involuntarily erect barriers against the recruitment, retention, and career progression of women. The fact that overt discrimination has been replaced with less obvious, primarily implicit preconceptions and prejudices against women makes gendered working circumstances even more difficult to change (eg, women have less career motivations, an assumed lack of confidence, and less ambition to take on leadership positions) [5]. Evidence suggests female researchers were disproportionately affected by the COVID-19 pandemic in terms of unequal distribution of childcare, elderly care, and other kinds of domestic and emotional labor reflected by a sudden drop in the number of female authors of scientific publications [6].

The National Surveys of Student and Staff Experiences of Sexual Violence and Sexual Harassment in Higher Education revealed over one-quarter of HEI staff did not feel safe from sexual harassment and 60% reported sexist hostility in the workplace. Among students, one-third or less felt safe socializing at night on campus or in the local community. In total, 66% of students reported experiencing sexist hostility [7].

A comprehensive review of state interventions to tackle gender inequality in Irish HEIs in Ireland from 2014 to 2019 indicated the ongoing presence of barriers impacting women’s opportunity to occupy academic or managerial positions of formal power [8]. The authors acknowledge that the impact of several state interventions is not yet clear; however, they advocate for the continuation of a multifaceted approach, which addresses this issue at “the state (macro), the HEI (meso), and the situational (micro)-level simultaneously.” To address and overcome gender inequality in the higher education environment, Acai et al [9] posit that change needs to occur from day 1 of the student’s academic experience. Gender equality and fairness training in third-level institutions are important steps toward creating a more inclusive and equitable learning environment for all students, regardless of their gender identity. Promoting gender equality and fairness in higher education is a complex issue that requires a multifaceted approach. Barriers contributing to the shortage of women in academics and academic leadership are numerous, including a shortage of role models and mentors [10]. Training is a critical tool for addressing gender inequality and promoting gender equality in higher education. The importance of gender-sensitive training material and nondiscriminatory education and training has been highlighted [11]. Appropriate and timely training has the potential to help students, faculty, and staff to recognize and address unconscious biases, stereotypes, and discriminatory practices that may exist within the institution. It can also help to promote a culture of respect, dignity, and mutual understanding among members of the campus community.

A recent study highlighted the gender inequality that exists in student leadership, demonstrating that although females made up 55% of the student body, they represented only 33% of the leadership in student organizations [12]. The Irish Higher Education Authority has emphasized a need to train both male and female student leaders in gender equality to increase awareness and nurture competences in order to actively address gender inequality. Such training will have an impact on the wider HEI community [3]. According to the European Institute for Gender Equality, gender equality training should enable and empower participants to (1) define and understand gender equality principles, (2) identify gender inequalities in their field, (3) be aware of gender in their planning and policy implementation, (4) monitor progress, and (5) review and assess their work from a gender perspective. Developing competence in gender equality means identifying and changing gender stereotypes and gendered roles, thereby creating a more equal society [13].

Similarly, UN Women identifies gender-equality competence as possessing the “awareness, knowledge, commitment and capacity necessary to incorporate gender perspectives into substantive work among professional staff.” At the heart of gender equality training lies transformation; to realize gender equality is to transform power sharing, control of resources, and decision-making to promote equal gender relations [14]. According to UN Women, gender equality training is not an event but a process. European Institute for Gender Equality and UN Women emphasize the necessity of *gender mainstreaming*, whereby individuals are provided with tools and training in gender equality competence-development and awareness-raising, but gender equality is simultaneously realized at an institutional and legal level [8,9].

At the heart of UN Women’s approach to gender equality training is the theory of change, which acknowledges that training plays an integral role in enhancing participants’ knowledge, motivation, and ability to promote gender equality, but that a long-term institutional commitment is also necessary to realize change. To optimize gender equality training, UN Women advises using Myra Marx Ferree’s Knowledge, Desire, and Ability model. Applying this approach recognizes that participants require all 3 components in order to enact change, including a knowledge and understanding of gender equality principles and gender inequalities, the desire and motivation to implement changes and address gender inequalities, and the tools and competences required to bring about change. The theory of change also encompasses engaging in reflexive practices to develop an awareness of one’s own thinking and behavior to become a self-reflexive learner and to foster gender equality over time. UN Women notes that the theory of change should be recognized as both a process and a product in empowering people and institutions to bring about gender equality [14].

### Study Aims

A preliminary literature review indicates that multiple gender equality–based training programs or initiatives for students have been designed and evaluated in second- and third-level education settings. Examples of educational activities undertaken include the delivery of didactic teaching, participation in a face-to-face collaboration project, site visits, case studies, and coaching [15-17]. Yet, our initial database searches indicated that, to date, a comprehensive review collating and synthesizing the available evidence on gender equality training for third-level students has not yet been carried out. Our review seeks to fill this gap.

This review will form part of the LIBRA study, which uses simulation techniques to develop a gender equality leadership training program for student leaders in higher education. This project is guided by the Irish Higher Education Authority’s Gender Equality Task Force recommendations under *integrating the gender dimension into teaching and learning by embedding the gender dimension into undergraduate or postgraduate education* [18]. In carrying out a review, we aim to identify and explore the existing literature on gender equality training and gender equality interventions for third-level students, with a particular emphasis on training content, methodology, and outcome evaluation. Second, the review seeks to construct a consensus on gender equality–enabling skills and leadership skills necessary to foster gender equality.

### Study Design

We considered a variety of approaches for analyzing the existing literature and deemed a scoping review most suitable for the requirements of this study. Arskey and O’Malley [19] list four principal reasons for conducting a scoping review, including (1) to explore the extent, range, and nature of available research on a specific topic; (2) to summarize and disseminate findings from the existing literature; (3) to identify a potential need for a systematic review; and (4) to identify gaps in the research literature. In line with these reasons, a scoping review will provide an appropriate methodology to explore the extent and nature of the existing research on gender equality training for third-level students. In designing the LIBRA gender equality simulation program, it will be essential that this process is informed by an evidence-based approach and uses the most up-to-date research regarding gender equality training in higher education. Leadership plays an integral role in the LIBRA program in order to empower student leaders to promote gender equality. This review also seeks to identify potential gaps in the literature regarding gender equality leadership training for third-level students.

Peters et al [20] explain that a scoping review is useful in clarifying key concepts and definitions in the literature. This will be particularly relevant within the LIBRA project to consolidate a definitive skills framework that outlines key competences required to promote gender equality and challenge gender inequality. Research questions informing a scoping review are typically wide in breadth, and researchers can draw on a diverse and heterogenous range of sources and study designs in carrying out the review. Conversely, the systematic review requires a detailed research question and generally includes a narrow range of quality-assessed studies [19-21]. This review will explore and present an overview of the existing literature on gender equality training, as opposed to ascertaining the efficacy of a specific intervention, so we deemed a scoping review the most suited approach for this study.

## Methods

### Overview

The review will be structured using Arskey and O’Malley’s [19], 5-stage framework, which involves (1) identifying the research question, (2) identifying relevant studies, (3) selecting the studies, (4) charting the data, and (5) collating, summarizing, and reporting the results. The scoping reviews’ systematic approach will also be informed by 3 additional studies, each of which has contributed depth and detail in advancing Arskey and O’Malley’s [19] original framework, including Levac et al [22], Peters et al [20], and Tricco et al [21].

### Identifying the Research Question

Tricco et al [21] list a variety of models to facilitate the development of a research question, including population, intervention, control, and outcomes tool and an adaption of the sample size, phenomenon of interest, study design, evaluation, research type tool. However, Peters et al [20] argue that the population, concept, and context mnemonic are more suited to crafting a research question suited to the exploratory and descriptive nature of the scoping review. The population, intervention, control, and outcomes model, in contrast, facilitates the development of focused and explanatory research questions more suited to a systematic review [20]. In forming the research question for this review, we selected the population, concept, and context approach: the population will include students in higher education, the concept will include gender equality training, and the context will encompass all HEIs.

The principal aims of this scoping review will be to (1) produce a descriptive overview of gender equality training and gender equality interventions for students in higher education or postsecondary education, including their core aims, key content areas, implementation methodology, and outcome evaluation, and (2) construct a consensus on skills and competences required to promote gender equality. A secondary aim of this study is to review the extent to which leadership is incorporated into gender equality training.

The principal research question for the review is: what is the current nature and scope of interventions promoting gender equality competences among students in higher education? The secondary research questions include (1) how is gender equality training delivered to students, and what are the key topics included in the intervention? (2) What specific skills and competences are taught to students to enable them to promote gender equality? (3) How is gender equality training evaluated? (4) To what extent is the concept of leadership included in gender equality interventions, and how are gender equality leadership skills fostered among students?

### Identifying Relevant Studies

The search strategy was designed in consultation with an experienced librarian. The search terms will comprise three thematic combinations, including (1) gender equality training, (2) HEIs, and (3) students, each separated with the Boolean operator AND. Within each thematic combination, search terms will be separated with the Boolean operator OR. Wildcards will be used to ensure the inclusion of plurals and variation in spelling across the search terms. The search will be limited to studies and other sources published between January 2011 and November 2021. Based on preliminary database searching, the majority of publications on gender equality training for second- and third-level students have been published in the last 10 years. Systematic searches will be carried out in 6 databases, CINAHL Plus and APA PsycInfo (accessed on Ebscohost), Embase, MEDLINE (Ovid), Web of Science (Science and Social Citation Indexes accessed on Clarivate), and Scopus (accessed on Elsevier). Textbox 1 provides an example of the search strategy, which will be applied. We will also carry out searches using MedEdPortal, MedEdPublish, and Open Grey to identify any gray literature that could further inform the review. The search will be limited to titles, abstracts, and keywords to ensure the search terms collate papers and sources whose focus relates to these topics.

**An example of search strategy applied for APA PsycInfo and CINAHL databases.**

1. TI (gender N2 training OR bias N2 training OR discrimination N2 training OR diversity N2 training OR equality N2 training OR inclusion N2 training OR sexuality N2 training) OR AB (gender N2 training OR bias N2 training OR discrimination N2 training OR diversity N2 training OR equality N2 training OR inclusion N2 training OR sexuality N2 training)
2. TI (gender N2 course$ OR bias N2 course$ OR discrimination N2 course$ OR diversity N2 course$ OR equality N2 course$ OR inclusion N2 course$ OR sexuality N2 course$) OR AB (gender N2 course$ OR bias N2 course$ OR discrimination N2 course$ OR diversity N2 course$ OR equality N2 course$ OR inclusion N2 course$ OR sexuality N2 course$)
3. TI (gender N1 program* or bias N1 program* or discrimination N1 program* OR diversity N1 program* or equality N1 program* OR inclusion N1 program* OR sexuality N1 program*) OR AB (gender N1 program* OR bias N1 program* OR discrimination N1 program* OR diversity N1 program* OR equality N1 program* OR inclusion N1 program* OR sexuality N1 program*)
4. TI (gender N1 awareness* or gender N1 bias or gender N1 equality OR gender N1 inclusion OR gender N1 equity OR sex N1 bias) OR AB (gender N1 awareness* or gender N1 bias or gender N1 equality OR gender N1 inclusion OR gender N1 equity OR sex N1 bias)
5. 1 OR 2 OR 3 OR 4
6. TI (“higher education” OR “third level education” OR “tertiary education” OR university* OR college$) OR AB (“higher education” OR “third level education” OR “tertiary education” OR university* OR college$)
7. TI (Undergraduate$ OR postgraduate$ OR student$) OR AB (Undergraduate$ OR postgraduate$ OR student$)
8. 1 AND 2 AND 3
9. LIMIT 8 to 2010-2021

Literature searches to be carried out in APA PsycInfo, CINAHL, Embase, MEDLINE (Ovid), Scopus, and Web of Science databases. Searches using MedEdPortal, MedEdPublish, and Open Grey will be carried out to identify gray literature to further inform the review. The search was limited to studies and other sources published between January 2011 and November 2021.

### Study Selection

The search will be carried out by the librarian and subsequently prepared for screening in an EndNote library (EndNote, version 20.2.1; Clarivate Analytics), and duplicates removed. The EndNote library will be exported to Rayyan (Rayyan), which will be used to collaboratively complete paper screening. Rayyan is a web-based tool designed to speed up the screening process of systematic reviews, scoping reviews, and other knowledge synthesis projects [23]. The principal investigator and research assistant will each carry out the title and abstract screening. Tricco et al [21] advise establishing an agreed standard for reviewing texts prior to commencing the formal screening process. We will use the following inclusion and exclusion criteria as our agreed framework for paper screening. In line with the study’s research questions, our inclusion and exclusion criteria will be applied as detailed in Textbox 2.

Throughout the course of the screening process, the inclusion and exclusion criteria will be adapted and developed in an iterative fashion. Researchers will resolve disagreements through discussion and develop a finalized list of abstracts to be reviewed. If consensus is not reached on particular sources, one of the coauthors will act as a third party in finalizing the selection decision. All authors will collectively review the first 10 texts screened for applicability to the research questions and use group discussion to add depth and detail to the inclusion and exclusion criteria. The remainder of the texts to be reviewed will be divided among 3 groups, with each group consisting of 2, 2, and 3 authors, respectively. A collaborative and discursive process will be applied to resolve disagreements at the full-text screening phase, and the collective work and consensus among each group will form the finalized list of full texts to be included in the scoping review. Throughout the screening phase, the authors will use a reflexive approach in reviewing papers and consolidating the inclusion and exclusion criteria, given the iterative nature of this process [20].

**The inclusion and exclusion criteria.**

**Inclusion criteria**

- Undergraduate or postgraduate students in higher education
- Interventions (eg, campaign or workshop) promoting gender equality awareness or gender equality competences:
  Interventions inside or outside of the academic curriculum
    - for example, project within a module or exercise within a module
    - for example, course or program external to course of study
  Papers or sources focusing on the experience of participation in an intervention
  Interventions do not have to focus solely on gender equality; interventions can also focus on other aspects of equality, diversity, and inclusion (EDI) training; as long as gender equality is included in the intervention, it can be included
  Interventions involving gender equality in terms of gender diversity (transgender, nonbinary, and gender-diverse students)
  Teacher training
- Third-level education institutions
- Published between January 2010 and September 2021
- Publications such as peer-reviewed literature and gray literature (dissertations, conference papers, corporate documents, government reports, preprints, proceedings, research reports, and periodicals)
- Any relevant papers cited in literature, systematic, and scoping reviews focusing on similar subject matters
- Studies in English

**Exclusion criteria**

- Primary or secondary students, higher education staff, general public
- Papers or sources focusing on:
  Measuring gender balance in higher education courses
  Strategies to promote greater equality among applicants to particular courses
  Experiences of gender or gender inequality among students
  Impact of gender on subject-specific competences, for example, programing or spatial awareness
  Recruitment or retention of female students in academic courses
  Gender equality in health care
  Mentorship programs
  Diversity or EDI programs that do not include a gender dimension
  Gender studies modules
  Papers focusing on a whole-of-campus institutional change
  Experiments on students' attitudes—unless these form part of a specified intervention.
- Papers referring to primary- or secondary-level school interventions, youth services, community services programs
- Published prior to January 2010
- Publications such as books, film reviews, and websites
- Studies for which no English translation is available

Reference lists of the included texts will be hand-searched for further relevant studies and sources, as will literature reviews identified in the database search. As per the exploratory nature of a scoping review, a variety of study designs and sources will be used to gather data, including empirical peer-reviewed research, periodicals, dissertations, conference abstracts, magazines, periodicals, and research reports. Appropriate gray literature will also be searched in the reference lists of selected texts. Quality appraisal will not form part of the screening and selection process. A scoping review principally seeks to identify, explore, and map existing literature on a given topic, so assessing the methodological and academic quality of the studies will not be applicable or necessary [20,22].

Finally, to optimize the comprehensiveness of the search, Levac et al [22] and Paez [24] recommend consulting experts in the field to inform and guide the review. We will liaise with several experts in the field of gender equality and leadership training via email. Experts will be asked to review the list of papers selected, provide feedback, and identify any further papers or ongoing studies that should be included. We aim to liaise with a variety of organizations in informing the review, including the UN Women Training Centre, the European Institution for Gender Equality, the Gender Equality Academy, RCSI Institute of Leadership, Technology University Dublin Equality, Diversity, and Inclusion Unit, University College Cork (UCC) Equality, Diversity, and Inclusion Unit, UCC Business School, the Irish Management Institution UCC, Aurora Management, 30% Club, and Advance Higher Education. The study selection process will be laid out using a flow diagram as per the PRISMA-ScR (Preferred Reporting Items for Systematic reviews and Meta-Analyses Extension for Scoping Reviews) Statement to make the search strategy and selection process transparent and auditable [19,21].

### Ethical Considerations

This review will involve the analysis of publicly available empirical research and the production of secondary data, so ethical approval will not be required.

## Results

### Overview

The extracted data will be collated, and a narrative summary will accompany the results to explain how the results relate to the reviews’ objectives. The findings will be discussed as they relate to higher education. Gaps and limitations of this literature will also be identified and presented.

### Charting the Data

A data-charting form will be used to collect key information from each study using Word (Microsoft Corp) documents and Excel (Microsoft Corp). MP developed the original data-charting form, which will be expanded and refined in an iterative fashion by all authors during the full-text screening process. The researchers will independently chart the data and update the data-charting table as they see necessary. Group discussion will be used to address disagreements and create the final standardized data-charting table. Arskey and O’Malley [19] explain that data charting differs from the data extraction process used in systematic reviews and meta-analyses, where data extraction is specific and may involve statistical techniques, data charting within scoping reviews involves a broader approach and includes narrative information, capturing how the study was carried out.

Peters et al [20] explain that the information documented in the data-charting table should align with the research aims and research questions of the review. As such, the data collected for this scoping review will include study details (author or year), location, context, type of source, study aims or intervention aims, participant details and sample size, research questions addressed, study design, gender equality theories used, intervention style and duration, intervention content, intervention methods and format, outcome evaluation measures, key findings, interesting observations, incorporation of gender equality and leadership, incorporation of intersectionality, and incorporation of transgender and gender-diverse inclusivity.

### Collating, Summarizing, and Reporting the Results

Levac et al [22] recommend dividing this final stage into 3 distinct components. To collate and summarize the results, we will use descriptive numerical summary analysis. The data will be mapped and presented in tabular format to make it easier to respond to the study questions. Basic descriptive analysis is both sufficient and the most suited approach of data analysis for the exploratory nature of the scoping review [21]. Finally, Levac et al [22] advise the researcher to consider the meaning of the findings in relation to the overarching purpose of the study. A narrative summary will accompany the results and will describe how the results relate to the review objective. Gaps and limitations of this literature will also be identified and presented.

## Discussion

### Principal Findings

HEIs have the potential to amplify gender equality through training for the betterment of society at large. We are undertaking this scoping review as part of a need assessment to provide the best evidence to inform intervention design and prioritize content for an educational program.

We aim to first synthesize the existing literature on training that supports gender equality in HEIs providing conceptual, theoretical, and evidentiary clarity on the current state of the art. Second, we aim to identify gaps and areas for future research to help define the research agenda for gender equality training in HEIs going forward.

### Study Dissemination

Research findings will be disseminated using a variety of mediums, including publication in a peer-reviewed academic journal and presentation of the findings at national and international conferences and other academic events. The findings will also be used to inform the pilot study for the LIBRA gender equality–based training program, with the goal of a subsequent national roll-out across Irish HEIs. Further afield, the findings will be available to be used by other universities or institutions of education to inform the development of gender equality training for students in higher education.

### Strengths and Limitations

To the best of our knowledge, this is the first review of the literature focusing on gender equality training and interventions for third-level students. The search strategy was comprehensively developed by all authors, with the support of an experienced librarian, which will facilitate a thorough and extensive database search. The university in which this study is being carried out is a medical and scientific institution, which will necessarily limit the full scope of databases available to the authors in carrying out the search. Following consultation with our librarian, hand-searching reference lists of the included papers and texts was considered sufficient to identify other important studies and gray literature in the event that they were not retrieved in the database search. We originally intended to incorporate Google and Google Scholar searching into the search strategy; however, given time limitations within the allocated funding period, we decided to focus specifically on academic databases when carrying out the search. This review is limited to papers written in English or which have an English translation available, which may bias the evidence.

It is also important to note that there may be a lack of relevant literature available in this particular field, given the niche topic on which this review will focus. However, this finding in itself may encourage further research on the topic as it would highlight a significant gap in the literature following a comprehensive search of the databases. Excluding studies focusing on gender equality in health care, mentorship programs, and gender studies modules may lead to the exclusion of useful information, both within the review itself and later informing the LIBRA study. Yet, following the preliminary database searching and screening, we concluded that these particular studies did not fully align with the research questions.

## Abbreviations

HEI: higher education institution
PRISMA-ScR: Preferred Reporting Items for Systematic Reviews and Meta-Analyses Extension for Scoping Reviews
UCC: University College Cork
